# The Membrane Glycoprotein M6a Endocytic/Recycling Pathway Involves Clathrin-Mediated Endocytosis and Affects Neuronal Synapses

**DOI:** 10.3389/fnmol.2017.00296

**Published:** 2017-09-20

**Authors:** Micaela D. Garcia, Karina Formoso, Gabriela I. Aparicio, Alberto C. C. Frasch, Camila Scorticati

**Affiliations:** ^1^Instituto de Investigaciones Biotecnológicas-Instituto Tecnológico de Chascomús, Universidad Nacional de San Martín, Consejo Nacional de Investigaciones Científicas y Técnicas Buenos Aires, Argentina; ^2^Instituto de Investigaciones Biomédicas, Universidad Católica Argentina Buenos Aires, Argentina

**Keywords:** PLP protein family, neuronal plasticity, traffic, endosomal compartments, Rab GTPases, glycoprotein M6a, synapse, clathrin-mediated endocytosis

## Abstract

Single point mutations or variations in the expression of the gene encoding the neuronal glycoprotein M6a have been associated with psychiatric disorders such as Alzheimer’s disease, depression and schizophrenia. In cultured neurons, M6a positively contributes to neurite extension, axon guidance, filopodia/spine outgrowth, and synapse formation. The endocytic processes of neuronal membrane proteins are linked to the differentiation, growth, signaling and plasticity of neurons. However, the roles of M6a and the precise mechanisms through which M6a internalizes and recycles back to the neuronal membrane are unknown. Here, by using a controlled *in vitro* assay, we showed that if 30–40% of M6a is endocytosed, the number of synapses in hippocampal neurons decreases. When re-establishing the levels of M6a at the cell surface, the number of synapses returned to normal values. M6a internalization involves clathrin-coated pits, probably by association between the adaptor protein 2 and the 251YEDI254 “tyrosine-based” motif located within the C-tail of M6a. Upon endocytosis, M6a is sorted to early endosome antigen 1- and Rab5-positive endosomes and then sorted back to the cell surface via Rab11-positive endosomes or to degradation via Rab7 and, finally LAMP-1-positive endosomes. Our results demonstrated that the levels of M6a at the cell surface modified the formation/maintenance of synapses, without altering the protein levels of synaptophysin or *N*-methyl-D-aspartate receptor type-1. This novel mechanism might be relevant during neuronal development, pruning and/or many of the neurological disorders in which the number of synapses is affected.

## Introduction

Endocytic recycling pathways are essential to preserve the proper composition of membrane proteins and for necessary molecules to return to their specific functions in suitable compartments (Maxfield and McGraw, 2004). Especially in neurons, endocytosis of neuronal growth factors, neurotransmitter receptors and scaffolding proteins regulates where and when signaling cascades are initiated. As a result, the endocytic recycling pathways of neuronal surface proteins are critical for many aspects of neuronal survival, axonal growth and guidance, dendritic branching, synapse formation and maintenance and cell migration (Farias et al., 2012; Cosker and Segal, 2014; Cousin, 2015; Britt et al., 2016).

**GRAPHICAL ABSTRACT:**
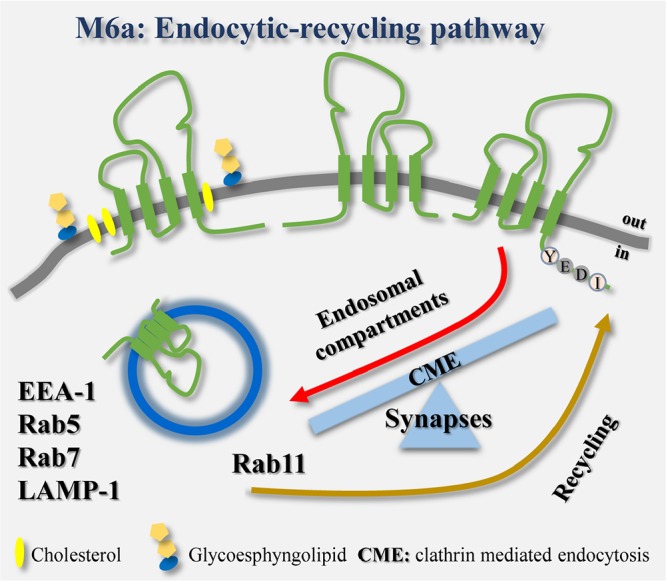
The endocytosis/recycling pathway of the glycoprotein M6a is linked to the formation and maintenance of synapses in hippocampal neurons.

The membrane glycoprotein M6a, together with proteolipid protein (PLP), DM20 and M6b belong to the tetraspan PLP family. M6a is a neuronal surface protein that promotes neurite and axonal outgrowth, filopodia/spine induction and synapse formation in primary neuronal cultures and neuronal cell lines (Lagenaur et al., 1992; Mukobata et al., 2002; Alfonso et al., 2005; Zhao et al., 2008; Sato et al., 2011a; Formoso et al., 2015a, 2016). However, the mechanisms by which M6a is sorted into different membrane compartments to assist neuronal plasticity remain unknown. In the case of PLP, the main protein of compact myelin, its trafficking and sorting are under neuronal control. PLP is required on the surface of oligodendrocytes during their differentiation and myelinogenesis. In cultured oligodendrocytes, neuron-glia communication regulates PLP clathrin-independent endocytosis and exocytosis (Winterstein et al., 2008). By contrast, in the absence of neural stimuli, PLP is internalized and accumulates in late endosomes/lysosomes as storage compartments (Winterstein et al., 2008; Roboti et al., 2009).

Clathrin-mediated endocytosis (CME) is a multi-step process that includes the formation, stabilization and maturation of clathrin-coated pits, through which surface receptors and other transmembrane proteins are sorted into clathrin-coated vesicles (Merrifield and Kaksonen, 2014; Cousin, 2015). Consequently, accessory and adaptor proteins such as adaptor protein 2 (AP-2) are recruited to the plasma membrane and then recycled back to the cytoplasm for subsequent cycles. In contrast, cargo receptors such as transferrin receptor (TfR) are constitutively internalized after ligand stimuli and sorted into the endosomal system. There, they can be targeted back to the plasma membrane to be reused or targeted to other compartments such as lysosomes (McMahon and Boucrot, 2011). Wu et al. (2007) and Liang et al. (2008) demonstrated that M6a boosts μ-opioid receptor endocytosis/recycling in MOPr-M6a co-expressing HEK293 cells exposed to agonist stimulation. In addition, by using polyclonal antibodies against the C-terminal of M6a in a co-immunoprecipitation assay followed by mass spectrometry analysis in rat hippocampal neurons, Fuchsova et al. (2015) showed that clathrin heavy chain co-precipitates with endogenous M6a. One of the key events leading to selective recruitment of cargo transmembrane proteins into different endosomal compartments is the interaction of their cytoplasmic tails with adaptor proteins (Bonifacino and Dell’Angelica, 1999). Regarding CME, several target signals have been identified. Tyrosine-based motifs YXXΦ (where X can be any amino acid and Φ is an amino acid with a bulky hydrophobic group) within the cytoplasmic tails have been identified as subjected to recognition by the mu2 subunit of AP-2 in many cargo proteins and receptors (Escudero et al., 2014; Liu et al., 2014).

Recently, the M6a gene expression levels have been linked to different neurological disorders such as Alzheimer’s disease, depression, mental retardation and schizophrenia (Boks et al., 2008; Greenwood et al., 2012; Gregor et al., 2014; Fuchsova et al., 2015; Lachen-Montes et al., 2016). Hence, M6a likely has an important role in neuronal plasticity and pathologies. Thus, the aim of this work was to characterize M6a internalization and its functional implications. Our results demonstrate that the M6a endocytosis/recycling pathway involves CME, probably by the association of the 251YEDI254 motif, within its C-tail, with AP-2. The decrease in M6a at the cell membrane is accompanied by a decrease in the number of synapses in cultured neurons. The refurbishment of M6a levels at the cell surface, via Rab11-positive endosomes, restores the number of synapses. However, about 20% of the endocytosed M6a is targeted to lysosomal compartments (LAMP-1-positive endosomes), where it might be subjected to degradation.

## Materials and Methods

### Animals

Sprague–Dawley pregnant female rats maintained at the Facultad de Farmacia y Bioquímica of the University of Buenos Aires (FFyB-UBA), Argentina, were used. All animal procedures were carried out according to the guidelines of the National Institutes of Health (publications No. 80-23) and approved by the Committee for the Care and Use of Laboratory Animals of the Universidad de San Martín (CICUAE-UNSAM No. 03/2015), Buenos Aires, Argentina.

### Reagents and Antibodies

Primary antibodies were: monoclonal anti-M6a rat IgG (1 μg/ml, anti-M6a mAb) and rat IgG2a-isotype control- (1 μg/ml, Medical and Biological Laboratories, Nagoya, Japan), polyclonal antibodies anti-synaptophysin rabbit IgG (1/300, Synaptic System GmbH, Goettingen, Germany), monoclonal anti-*N*-methyl-D-aspartate mouse IgG (NMDA)-receptor type 1 (R1) (1/500, Synaptic System), monoclonal anti-tubulin mouse IgG (1/1000, Sigma, Munich, Germany), polyclonal anti-M6a C-terminal rabbit IgG (1/1000), monoclonal anti-Rab5 (C8B1) rabbit IgG (1/1000, Cell Signaling), monoclonal anti-clathrin heavy chain rabbit IgG (1/700, Cell Signaling), monoclonal anti-LAMP-1 mouse IgG (H4A3 clone) (1/100, Hybridoma Bank, Iowa University, United States), monoclonal anti-early endosome antigen 1 (EEA1) mouse IgG (1/700, BD Biosciences, United States) and anti-EEA1 rabbit IgG (1/1000, Cell Signaling) and polyclonal anti-GFP rabbit IgG (1/500, Invitrogen). Preabsorbed secondary antibodies were: Alexa 633 goat anti-mouse IgG (1/1000, Invitrogen, Leiden, the Netherlands), Alexa 647 goat anti-mouse IgG (1/1000, Invitrogen), Alexa 568 goat anti-rabbit IgG (1/1000, Invitrogen), Alexa 568 donkey anti-goat IgG (1/1000, Invitrogen), Rhodamine conjugate goat anti-rat IgG (1/1000, Jackson Laboratories, USA) and Alexa 488 goat anti-rat IgG (1/1000, Invitrogen). Reagents were: LysoTracker^®^ Deep Red (75 nM-1 μM, Invitrogen) and Transferrin Alexa 647 conjugate (Tf-647 50 μg/ml, Invitrogen). Methyl-β-cyclodextrin (5 mM), monensin (50 μM), filipin (5 μg/ml) and cycloheximide (10 μM) were all purchased from Sigma.

### Plasmids

The expression vectors used were: M6a wild type (wt) and Y251A mutant tagged with the green fluorescent protein (GFP) or red fluorescent protein (RFP) (Formoso et al., 2015a). Rab5-GFP and Rab7-GFP (Escudero et al., 2014) and Rab11-GFP (Lazo et al., 2013) were generously supplied by Dr. Francisca Bronfman (Pontificia Universidad Católica de Chile). LAMP1-GFP tagged (Rodriguez-Walker et al., 2015) was kindly supplied by Dr. Jose Luis Daniotti (Universidad Nacional de Córdoba, Argentina) from Dr. Juan S. Bonifacino‘s lab (NIH, Bethesda, MD, United States). The identity of all constructs was verified by DNA sequencing.

### Hippocampal Neurons and HEK293 Cultures

Dissociated neuronal cultures were prepared from embryonic day 19 fetal rat hippocampi, as previously described (Formoso et al., 2015a). Briefly, tissues were treated with 0.25% trypsin in Hanks’ solution for 15 min at 37°C. A single-cell solution was prepared in Neurobasal medium (NB, Invitrogen) containing 200 mM glutamine (Sigma), 100 units/ml penicillin, and 100 μg/ml streptomycin (NB1X) with 10% (v/v) horse serum (Gibco, Walkersville, MD, United States). Cells were seeded either on coverslips or six-well plates both coated with 0.1 mg/ml poly-L-lysine hydrobromide (Sigma) and 20 mg/ml laminin (Invitrogen). After 2 h, the medium was changed to NB/N2 medium (NB1X with 1 g/l ovalbumin; N2 and B27 serum-free supplements from Invitrogen). To perform functional analysis, neurons were cultured at a low density of 7000 cells per well in a 24-well plate for 15 days *in vitro* (DIV). To analyze the percentage of M6a endocytosis by flow cytometry, neurons were cultured at a high density of 1.5 × 10^6^ cells per well in a six-well plate (as described later). To analyze the co-distribution of endogenous M6a or M6a-GFP/RFP with the different endosomal markers, neurons were transiently transfected with Lipofectamine 2000 (Invitrogen) according to the manufacturer’s instructions.

Human embryonic kidney cells (HEK293) were cultured in Dulbecco’s modified Eagle’s medium (DMEM, Gibco) supplemented with high glucose 0.35% (m/v), L-alanine L-glutamine (200 mM, GlutaMAX, Invitrogen), 10% (v/v) fetal bovine serum (FBS, Gibco) and gentamicin 5 mg/ml. Cells were seeded on coverslips in 24- or 6-well plates and then transiently transfected with M6a-GFP/RFP and/or with the different endosomal markers by the polyethylenimine (PEI, FFyB-UBA) method, according to the manufacturer’s instructions. Briefly, 1 μg DNA mixed with 1.5 μl of PEI (25 mM) and 50 μl of OPTIMEM were incubated for 8 min at room temperature. Then, 200 μl of supplemented DMEM was added to the mixture and dropped into each well. Cells were incubated for 2 h at 37°C and then the transfection mixture was replaced by 500 μl of fresh supplemented medium. Sixteen hours later, HEK293 cells were subjected to the antibody internalization assay (AIA) or immunolabelling as described later.

Colocalization studies were carried out with an Olympus FV1000 confocal laser microscope attached to an Olympus IX81 inverted microscope (Olympus America, Melville, NY, United States). Confocal images were acquired as described below.

### Antibody Internalization Assay (AIA)

**FIGURE 1:**
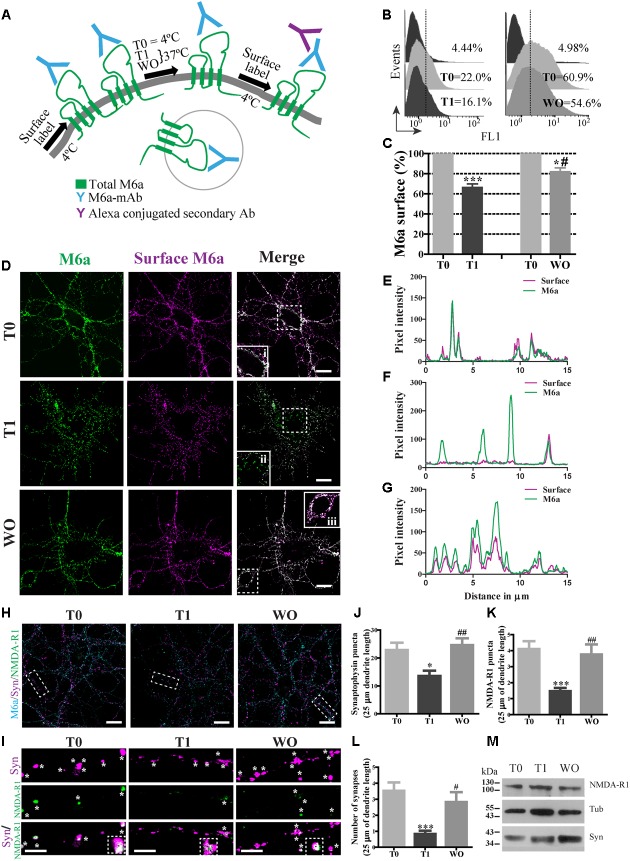
M6a internalization reduces the number of synapses in hippocampal neurons. **(A)** Scheme of the monoclonal antibody internalization assay (AIA) in which T0 and T1 represent cells exposed to the monoclonal antibody (M6a mAb) or isotype control (1 μg/ml) for 1 h at 4°C or 37°C respectively, and WO represents cells that were subjected to antibody uptake followed by washout for 20 h in cultured. Alexa 488 conjugate secondary antibodies bound to the remaining complexes (M6a/M6a mAb) at the cell surface. **(B)** Representative histograms of the fluorescence intensity (FL1) versus the number of neurons (events) subjected to the AIA. **(C)** Three independent experiments were analyzed and the percentage of M6a at the cell surface was relativized within each T0 and plotted. Statistically significant differences was found [*F*(3,8) = 38.26, *p* < 0.0001; one way ANOVA] between T0 vs. T1 [^∗∗∗^*p* < 0.001 (*n* = 3); Bonferroni *post hoc*]; T0 vs. WO and T1 vs. WO [^∗^/^#^*p* < 0.05 (*n* = 3); Bonferroni *post hoc*]. **(D)** Representative images from confocal microscopy of neurons at 15 DIV subjected to M6a mAb uptake for each condition. Total M6a was labeled with the secondary antibody Alexa 488 (shown in green) and the M6a remaining at the cell surface after the AIA was labeled with the tertiary antibody Alexa 568 (shown in magenta) under non-permeabilized conditions. Colocalization of green and magenta channels is shown in white. **(E–G)** RGB profiles of the soma insets at T0 (i), T1(ii) and WO (iii). Scale bar: 15 μm. Inset: 25 × 25 μm. **(H)** Representative images from confocal microscopy of 15 DIV hippocampal neurons subjected to AIA and then fixed and labeled for synapse quantification. Endogenous M6a is shown in cyan, the pre-synaptic marker synaptophysin, syn, is shown in magenta, and the post-synaptic marker NMDA-R1 is shown in green. Scale bar: 15 μm. Dashed rectangles show the 25 × 10 μm inset region that was analyzed. **(I)** Representative images of segments of 25 μm of dendrite length for each condition. White asterisks or black squares represent detectable puncta by the Puncta Analyzer (ImageJ) of syn, NMDA-R1 and the colocalization of syn/NMDA-R1. Scale bar: 5 μm. **(J,K)** Puncta Analyzer quantification of clusters of each synaptic marker along 25 μm of dendrite length. **(L)** Cluster colocalization of syn/NMDA-R1 as average number of synapses. The data show a representative experiment of three independent experiments in which at least 20–25 neurons (three segments per neuron) were analyzed for each condition. Significant differences were determined using one way ANOVA, Syn puncta: *F*(2,87) = 6.38, *p* = 0.19; NMDA-R1 puncta: *F*(2,87) = 9.22, *p* < 0.0001 and Synapses: *F*(2,87) = 8.51, *p* < 0.0001. Bonferroni *post hoc* analysis reveals significant differences between: T0 vs. T1 ^∗^*p* < 0.05 (*n* = 30); T1 vs. WO ^##^*p* < 0.001 (*n* = 30) in Syn puncta; between T0 vs. T1 ^∗∗∗^*p* < 0.001 (*n* = 30); T1 vs. WO ^##^*p* < 0.001 (*n* = 30) in NMDAR-1 puncta and T0 vs. T1 ^∗∗∗^*p* < 0.001 (*n* = 30); T1 vs. WO ^#^*p* < 0.05 (*n* = 30) in synapses. **(M)** Samples of each condition were subjected to SDS-PAGE followed by Western blotting with anti-syn, anti-NMDA-R1 and alpha-tubulin (loading control). The expected monomeric band of each protein is indicated.

HEK293 cells or primary hippocampal neurons were incubated on ice for 30–45 min with anti-M6a mAb (1 μg/ml) or a non-related monoclonal antibody of the same isotype of anti M6a-mAb (rat IgG2a –isotype control- 1 μg/ml) in complete medium (**Figure 1A**). Afterward, in the case of endogenous M6a in neurons, cells were incubated with Alexa 488 anti-rat IgG for 30–45 min on ice. Cells were either maintained on ice (T0) or incubated at 37°C for 30 min or 1 h (T30 or T1) to either prevent or allow the endocytosis of the complexes. Subsequently, cells were either incubated with tertiary antibodies to detect the residual M6a at the cell surface with Alexa 568 anti-goat IgG (for endogenous M6a) or with Alexa 488 anti-rat IgG or Rhodamine anti-rat IgG (for M6a-overexpressing cells). To assess whether M6a recycles back to the cell surface, cells were washed out (WO) after antibody uptake and maintained in culture for 4 or 20 h at 37°C before incubation with tertiary antibodies. In the case of HEK293 cells, cells were WO for 4 h in the presence of cycloheximide 10 μM to prevent new protein synthesis. Tf-647 was added to the endocytosis medium to track CME and the recycling endosomes. LysoTracker^®^ Deep Red was added to the endocytosis medium to characterize acidic compartments. Lastly, cells were washed and fixed with 4% paraformaldehyde (PFA), immunostained and mounted for subsequent confocal analysis of M6a internalization.

### Flow Cytometry

Neurons at 1 DIV or M6aGFP-HEK293 cells were seeded in six-well plates and 2 h later subjected to the AIA. Then, cells were mechanically harvested with a scraper in a minimum volume of pre-chilled PBS and pelleted at 500 g at 4°C. Cells were resuspended in a 2% PFA-PBS solution and incubated for 20 min at room temperature. Then, one volume of PBS was added to the tube to dilute the PFA to 1%. Fixed cells were analyzed by flow cytometry to determine the percentage of fluorescence in the FL1 channel (green fluorescence) by using a cell FlowMax cytometer PASIII (Partec, Munster, Germany) with FlowJo software v10.0.7. In neurons, the values obtained were relative to the control T0, which represents the total M6a at the cell surface taking into account the neuron stage (T0-1 DIV). In the case in which the WO cells were maintained for 20 h in the incubator, the values of WO cells were relative to the control at 2 DIV (T0-2 DIV), which represents the total M6a at the cell surface at this stage. All the data were expressed as % of M6a at the cell surface (Jung et al., 1996; Kawauchi et al., 2010). Regarding M6aGFP-HEK293 cells, flow cytometry analysis was used to determine the total amount of M6aGFP per cell in each condition. A batch of cells were stained with 7-Aminoactinomycin D (7-AAD, Biolegend, United States) to analyze differences in cell viability among the groups. The quantification of the incorporation of 7-AAD by flow cytometry in cells at TO and T1 were equivalent to the intact neurons.

### Synapse Assays

Synapse assays were carried out at a low density of neurons (7000 neurons/well) in 24-well plates. Neurons at 15 DIV were subjected to the AIA followed by methanol fixation [90% methanol and 10% buffer MES (100 mM MES pH 6.9, 1 mM EGTA, 1 mM MgCl_2_) for 5 min at 4°C]. Then, cells were washed with PBS-Tween (0.1%) for 5 min. FBS-Triton X-100 (FBS 10%, Triton X-100 0.1% diluted in PBS) was used as a first blocking solution. The second blocking solution was BSA 3% diluted in PBS for 20–30 min at 25°C. Both blocking solutions were centrifuged at maximum velocity for 10 min before use. Both primary antibodies (anti-NMDA-R1 and anti-syn) were diluted in PBS-BSA 1% solution and centrifuged for 10 min at maximum velocity and afterward incubated at 4°C overnight. Cells were washed and blocked again with 3% BSA and 10% FBS, Triton X-100 0.1% PBS for 1 h at 25°C followed by incubation with the secondary antibodies Alexa 633 anti-mouse IgG and Alexa 568 anti-rabbit IgG. Secondary antibodies were diluted at 1:1000 in 1% BSA in PBS previously centrifuged for 10 min at maximum velocity. Coverslips were mounted in Fluorsave^®^ (Calbiochem). Fluorescence images were acquired with a confocal microscope.

### Image Analysis

Cells were imaged at 60 X objective lens with a numeric aperture of 1.42 on an Olympus FV1000 confocal microscope. We setup the Olympus Fluoview v3.1a software to acquire the images with a 4–10 μs/pix of dwell time. We manually adjusted the laser energy setting (HV, gain and offset) by using slides stained only with the secondary antibodies to determine the threshold of background signal, which was applied to each image of the experiment. The saturation level was maintained by “AutoHV” mode. Images were taken in raster scan mode satisfying the Nyquist criterion, pixel size was 2–3 times smaller than the object. For colocalization HEK293 cells images were taken at 4X magnification and the image size was 1024 × 1024 pixels (pixel size was 0.051 μm/px). Neuron images were taken at 2X magnification and the image size was 1600 × 1600 pixels (pixel size was 0.066 μm/px).

Synapse formation in hippocampal neurons was measured by colocalization of puncta, along 25 μm of dendrite length, between pre- and post-synaptic markers in approximately 20 or 30 neurons per condition, using three dendritic regions per neuron. The selected neurons were at least two cell diameters away from their nearest neighbor. All experiments were carried out under blind conditions to the examiner. Colocalization of puncta were determined using the plugin Puncta Analyzer from Image J (NIH) (Ippolito and Eroglu, 2010). Briefly, 2–3 regions of interest from each neuron (25 μm of dendrite length) were selected and then background values were subtracted. The threshold was adjusted manually for each channel. This step was controlled by using the same threshold for each channel in the same experiment. The minimum puncta size was set to 4 pixels. At least three independent experiments were performed.

The quantification of the colocalization between endocytosed M6a with endocytic markers in HEK293 cells were performed using the ComDet Image J plugin v0.3.6.1(Esteves da Silva et al., 2015).^1^ Particles were detected in both green and red channels independently at approximated particle sizes of 4 pixels with sensitivities of signal/noise ratio equaling 4–5. Colocalization was determined based on a maximum distance between two particle centers of 4 pixels and expressed as percentage.

The digital images were processed with ImageJ software^2^.

### Quantification of M6a Internalization

M6a- and/or Y251A-expressing HEK293 cells were subjected to the AIA as above. M6a internalization was quantified by generating grayscale 8-bit conversion of confocal images from one image plane from the z-stack and analyzing the resulting data from these images. One region of interest was defined in a cell external perimeter and the total cell fluorescence (TF) intensity measured. Another region of interest was defined in the cytoplasm and the cytoplasmic fluorescence (CF) intensity measured. The ratio of TF/CF^∗^100 of the fluorescence corresponding to the transfected protein channel was plotted as a percentage of M6a or the Y251A mutant at the cell surface (Miserey-Lenkei et al., 2001; Leterrier et al., 2004).

### Western Blotting

Whole cell lysates were prepared in the presence of protease cocktail inhibitor and phosphatase cocktail inhibitors. Samples containing an equal amount of proteins from neurons or an equal amount of cells transfected with M6aGFP for each condition were separated with a 10% SDS/PAGE. Proteins were transferred into nitrocellulose membranes (Millipore) in a tank blot apparatus (Bio-Rad Laboratories). The membranes were blocked in Tris-buffered saline (TBS) containing 5% of non-fat dried milk for 1 h at room temperature and incubated with primary antibodies diluted in 1% BSA-PBS overnight at 4°C. The blots were washed with TBS supplemented with 0.2% Tween-20 (T-TBS) and incubated with HRP-conjugated secondary antibodies for 2 h at room temperature. The antigen-antibody complexes were detected according to a standard Enhanced Chemiluminescence Western blotting protocol using Super Signal Chemiluminescent Substrate (Pierce) and X-OMAT films (Kodak).

### Statistical Analysis

Calculations were performed with GraphPad Prism 5.0 (San Diego, CA, United States) and data are expressed as mean + SEM. Student’s *T*-test or one-way ANOVA followed by Bonferroni post-test calculations were used as appropriate and referred in the legend of each figure. The results were considered significant when *p* < 0.05.

## Results

### Monoclonal Antibody against M6a-EC2 Mediates the Internalization of M6a in Neurons

The monoclonal antibody against the main external loop (EC2) of M6a (M6a mAb) has been used as a neutralizing antibody that blocks M6a neurite extension in neurons and neuronal cell lines (Lagenaur et al., 1992; Sato et al., 2011a; Formoso et al., 2015a). We therefore examined whether M6a mAb mediates M6a internalization in cultured neurons. For that purpose, hippocampal neurons were bound with 1 μg/ml of M6a mAb or isotype control in NB media for 45 min at 4°C. The medium was replaced and neurons remained at 4°C (T0, steady state) or cultured at 37°C (T1) to induce M6a endocytosis. Either 1 h (T0 and T1) or 20 h later (WO), the residual M6a present at the cell surface was labeled with secondary antibodies Alexa 488-conjugated and analyzed by flow cytometry (antibody internalization assay -AIA-, **Figure 1A**). **Figure 1B** shows a representative histogram plot of the fluorescence intensity (FL1) versus the number of neurons (events) detected in one independent experiment. The FL1 of cells treated with the isotype control was used to determine M6a-positive and M6a-negative cells (∼4–5% of the total cells assessed). The incubation for 1 h at 37°C with M6a mAb clearly reduced the percentage expression of M6a at the cell surface (by ∼40%) compared with the neurons that remained at 4°C. In contrast, neurons subjected to the WO treatment partially recovered the percentage expression of M6a at the cell surface compared with the internal control (∼80% cell surface expression, **Figure 1C**). We next analyzed M6a subcellular destination after M6a mAb treatment by confocal analysis. **Figure 1D** shows representative images of neurons at 15 DIV subjected to the AIA in which total M6a was labeled with secondary antibodies. Afterward, M6a internalization was induced for 1 h at 37°C and subsequently labeled with tertiary antibodies, which are directed against the species of the secondary antibody, to differentiate only the M6a at the cell surface. In cells kept at 4°C during the assay (T0), the distribution of endogenous M6a was restricted to the cell surface as shown by a complete overlap of M6a secondary and tertiary antibodies labeling at the soma (inset 1D*i* and **Figure 1E**). In cells incubated at 37°C (T1), internalized M6a, visualized as puncta, was present in the soma and dendrites (inset 1D*ii* and **Figure 1F**). Here again, in neurons subjected to WO for 20 h, the surface localization pattern of endogenous M6a was similar to that observed in T0 cells and could be seen in the inset and in its RGB profile (inset 1D*iii* and **Figure 1G**). Indeed, endogenous M6a recovered its subcellular localization 4 h after removal of the monoclonal antibody (Supplementary Figures S1A,B). These data suggest that M6a mAb mediates M6a internalization and that, after the WO period, M6a returns to the cell surface, however, a role of degradation of M6a in this process cannot be excluded.

### M6a Internalization Reduces the Number of Synapses in Neurons

We therefore wanted to determine whether M6a internalization might affect synapse maintenance in neurons. Low-density cultured neurons at 15 DIV were subjected to the AIA and then fixed and labeled for synapse quantification by using Puncta Analyzer as previously reported (Formoso et al., 2016). **Figure 1H** shows a representative image of 15 DIV neurons, where endogenous M6a was labeled with cyan, syn with magenta and NMDA-R1 with green for each condition (T0, T1 and WO). Accordingly, we quantified the number of clusters of both markers and the colocalization puncta between syn and NMDA-R1 as a synapse by using Puncta Analyzer plugin of the ImageJ software. **Figure 1I** shows a representative image of 25 μm of dendrite length for each condition in which the synaptic clusters are shown in white asterisks and the colocalization cluster of both markers are shown in white asterisks and black squares. M6a internalization significantly decreased the number of clusters of both markers (**Figures 1J,K**) and the number of synapses (**Figure 1L**) compared with T0 neurons. In contrast, the WO treatment (20 h) recovered the number of puncta of both markers and the number of synapses at the neuron levels at T0. Although the endogenous M6a returned to the cell surface 4 h after removal of the monoclonal antibody, this time duration was not sufficient to restore the number of synapses per neuron (Supplementary Figures S1C,D). We further tested the possibility that M6a internalization might modify the protein levels of syn and NMDA-R1. Therefore, 1 × 10^6^ neurons were cultured for 15 days and subjected to the AIA followed by Western blot. However, none of the protein levels of the synaptic markers were modified during the assay (**Figure 1M**). These results allow us to speculate that M6a is required at the cell surface for synapse formation/maintenance and that the decrease in puncta of syn and NMDA-R1 is not due to a decrease in the protein levels.

### Clathrin Participates in M6a Endocytosis in Neurons and HEK293 Cells

**FIGURE 2:**
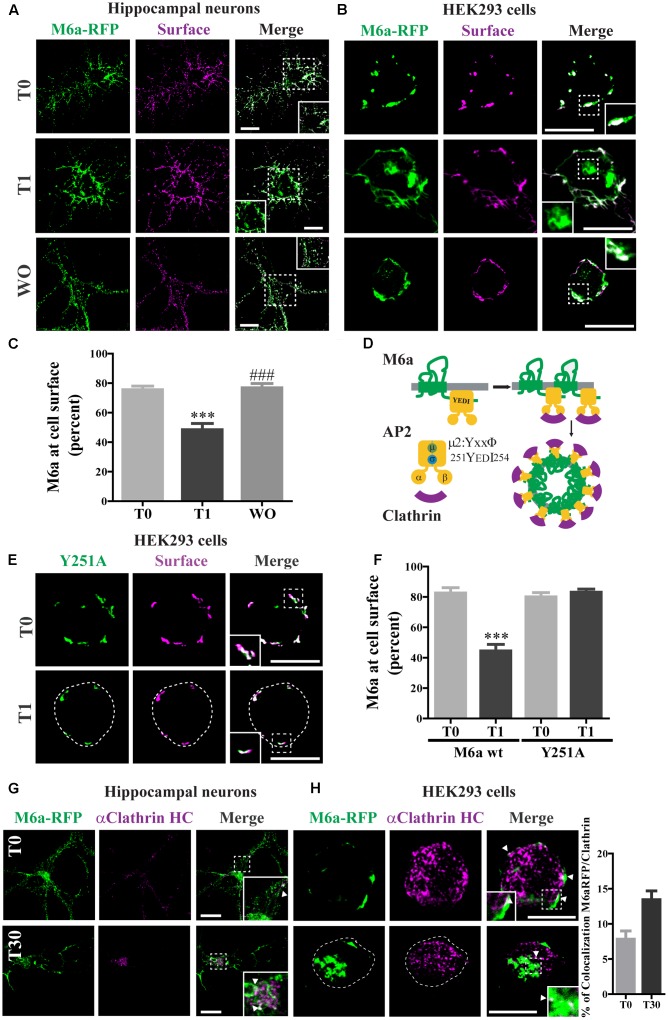
Clathrin participates in M6a endocytosis in neurons and HEK293 cells. **(A,B)** Representative images from confocal microscopy showed either **(A)** M6aRFP-overexpressing neurons at 15 DIV (shown in green) or **(B)** M6aRFP-overexpressing HEK293 (shown in green) subjected to the antibody internalization assay (AIA). After the AIA, cells were WO with fresh medium with or without cycloheximide for 4 h (WO). Alexa 488-conjugated secondary antibodies were used to detect the M6a remaining at the cell surface (shown in magenta). In the case of colocalization, the overlapping is shown in white. **(C)** The percentage of M6a at the cell surface was quantified in three independent experiments in M6aRFP-overexpressing HEK293 subjected to the AIA, as described in the Section “*Materials and Methods.”* Significant differences were determined using one way ANOVA followed by Bonferroni test [*F*(2,46) = 21.04, *p* = 0.02 between T0 vs. T1 ^∗∗∗^*p* < 0.001 (*n* = 20)]. **(D)** Scheme of M6a at the cell membrane and its internalization via clathrin-coated pits. M6a (in green) shows its C-tail tyrosine motif, YxxΦ, which may allow the interaction with adaptor protein 2 (AP-2, in yellow) through its mu domain, and this complex can also associate with clathrin (in magenta). **(E,F)** Y251A-GFP-overexpressing HEK293 cells were subjected to the AIA. **(E)** Representative confocal microscopy images of Y251A-HEK293 cells in which total expressed protein is shown in green and that remaining at the cell surface is shown in magenta **(F)**. Three independent experiments were quantified as described in **Figure 3C**. Significant differences were determined by Student‘s *t-* test (*t*16 = 6.89, *p* = 0.53) between T0 (M6a wt) vs. T1 (M6a wt) [^∗∗∗^*p* < 0.0001 (*n* = 9)]. **(G,H)** Representative images from the AIA either in M6aRFP-overexpressing neurons or HEK293 cells (shown in green) labeled with monoclonal anti-clathrin heavy chain (1:500, shown in magenta) are shown in **(G**,**H)** respectively. The overlapping signal of both channels is shown in merge columns and in white. White arrows indicate colocalization regions. The percentage of colocalization between endocytosed M6a with clathrin in HEK293 cells was plotted in H (for statistical data see Supplementary Table S1). Scale bars: 15 μm. Inset for HEK293: 4 × 4 μm; inset for neurons: 10 × 10 μm.

The cytosolic tails of the transmembrane proteins that possess “tyrosine-based” linear motifs (YXXΦ or [FY]XNPX[YF]) as well as “dileucine-based” motifs ([DE]XXXL[LI]) can be subject to CME recognized by AP-2 (Traub and Bonifacino, 2013). We used the eukaryotic linear motifs (ELM) database^3^ to predict the presence of endocytosis signals within the amino acid sequence of M6a (Dinkel et al., 2016). Four predicted sorting signals that interact with AP2 and are responsible for the internalization of M6a via clathrin-coated pits are summarized in **Table 1**. All the PLP family members have two external loops -minor EC1 and major EC2-, one intracellular loop -IC-, four transmembrane domains, and the N- and C- terminal regions in the cytoplasm. The sequences predicted by the ELM database placed the tyrosine-based motifs at the EC1, IC, and EC2 and in the C-terminal of M6a, respectively (**Table 1**). However, as described above, the sequence 251YEDI254 in the cytoplasmic C-tail of M6a is suitable for AP-2 complex subunit mu2 interaction and sorting into clathrin-coated endosomes, as shown in **Figure 2D**. We further investigated whether clathrin is involved in the M6a endocytosis/recycle pathway in M6a-overexpressing hippocampal neurons and HEK293 cells. HEK293 cells -which do not express endogenous M6a- have been widely used to track and compare the endocytosis/recycle pathways of many GPCRs and transmembrane proteins (Wu et al., 2007; Conti et al., 2009). For that purpose, we first assessed the AIA in both M6a-overexpressing neurons and HEK293 cells. Thus, hippocampal neurons at 15 DIV and HEK293 cells expressing the M6aRFP fusion protein were subjected to M6a mAb immunointernalization as described above. At T0, as in the case of endogenous M6a, confocal images exhibit almost complete overlap of total and surface M6a (**Figures 2A,B**, upper line). In contrast, at T1, cell somata showed punctate labeling of endocytosed M6a unlabelled with the secondary antibodies in both neurons and HEK293 cells (**Figures 2A,B**, middle line). As expected, the WO treatment (4 h) completely recovered M6a distribution at the cell surface, showing equal stain distribution between the overexpressed protein and the secondary antibodies labeled in neurons and HEK293 cells (**Figures 2A,B**, lower line). The ratio of (TF/CF)^∗^100 of the fluorescence corresponding to the transfected protein channel was plotted as a percentage of M6a at the cell surface in HEK293 cells (**Figure 2C**). The resulting quantification confirmed the internalization of M6a. Cells exposed for 1 h at 37°C with anti M6a mAb showed a significant decrease in the percentage of M6a on the cell surface compared with cells at T0. By contrast, M6a-HEK293 cells subjected to WO treatment totally recovered the percentage of M6a at the cell surface, equivalent to cells at T0. M6a-overexpressing cells exhibited the same endocytosis/recycling behavior as endogenous M6a (**Figure 1D** and Supplementary Figure S1A) and can therefore be used to track its cellular fate.

**Table 1:** Tyrosine-based motifs within the rat Gpm6a amino acid sequence.

Domain	Motif	Position	ELM name	ELM description	Cell compartment
EC1	YFEM	61–64	TRG-ENDOCITIC 2	Tyrosine-based sorting signal responsible for the interaction with mu subunit of AP complex	Plasma membrane, cytosol, clathrin-coated endocytic vesicle
IC	YGDF	114–117	TRG-ENDOCITIC 2	Tyrosine-based sorting signal responsible for the interaction with mu subunit of AP complex	Plasma membrane, cytosol, clathrin-coated endocytic vesicle
EC2	YMYF	153–156	TRG-ENDOCITIC 2	Tyrosine-based sorting signal responsible for the interaction with mu subunit of AP complex	Plasma membrane, cytosol, clathrin-coated endocytic vesicle
C-terminal	YEDI	251–254	TRG-ENDOCITIC 2	Tyrosine-based sorting signal responsible for the interaction with mu subunit of AP complex	Plasma membrane, cytosol, clathrin-coated endocytic vesicle

*Minor extracellular domain (EC1), intracellular domain (IC), major extracellular domain (EC2) and C-terminal domain (UniProtKB - Q812E9). Eukaryotic linear motif research database (ELM 2016)*.

**FIGURE 3:**
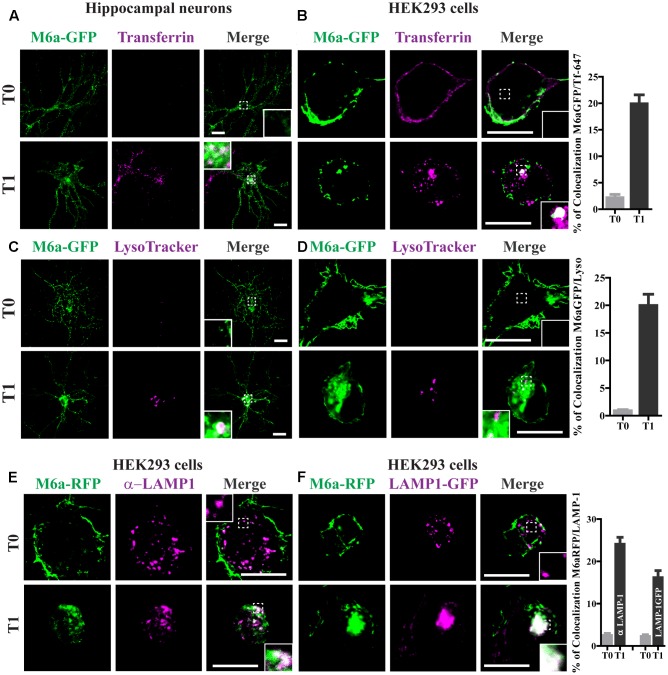
Endocytosed M6a is sorted to both recycling and degradative compartments. Neurons at 15 DIV and HEK293 cells were subjected to the antibody internalization assay (AIA) in the presence of transferrin 647 conjugated (shown in magenta, **A**,**B**) or deep red LysoTracker^®^ (**C**,**D**, shown in magenta). Representative confocal images from either neurons **(A,C)** or M6aGFP-overexpressing HEK293 cells **(B,D)**. **(E)** Representative images from M6a-HEK293 cells subjected to the AIA and labeled with anti-LAMP1 (shown in magenta). **(F)** Coexpressed M6aRFP/LAMP1-GFP cells subjected to the AIA. Conditions in which dots exhibited colocalization between M6a and the corresponding endocytic compartments are shown in white in the inset of merge images. The percentage of colocalization between endocytosed M6a with Tf-647, LysoTracker andLAMP-1 in HEK 293 cells were plotted in **(B,D,F)** respectively (for statistical data see Supplementary Table S1). T0 and T1 represent the steady state and 1 h after immunouptake respectively. Scale bar: 15 μm. Inset for neurons: 5 × 5 μm; inset for HEK293: 4 × 4 μm.

To evaluate whether the 251YEDI254 motif in the C-tail of M6a participates in the endocytosis/recycling pathway (**Figure 2D**) M6a wt and Y251A mutant were expressed in HEK293 cells and treated for 1 h with M6a mAb at 4°C (T0) and at 37°C (T1): (**Figures 2D**, **3E** and Supplementary Figure S2). The Y251A mutation totally abolished M6a internalization induced by the monoclonal treatment without interfering in its immunodetection, showing a complete overlap along the cell surface at T0 and at T1 (**Figure 2E**). M6a quantification at the cell surface of M6a wt vs Y251A mutant is shown in **Figure 2F**. As previously shown (**Figure 2C**), the presence of M6a wt at the cell surface in HEK293 cells was significantly reduced by the antibody treatment (T1). However, in Y251A-expressing cells, the presence of M6a at the cell surface exhibited no quantitative changes in any condition compared to M6a wt-expressing cells at T0. These results suggest that tyrosine at 251 seems to have a critical role in M6a endocytosis.

Finally, to test whether M6a endocytosis might occur via clathrin-coated pits, we determined the colocalization of M6a and clathrin in M6a-expressing neurons and HEK293 cells. In both cell types, clathrin showed punctate stained and colocalized with M6a at T0 and after 30 min of M6a immunointernalization (T30). At T0, colocalization was restricted to the cell surface and at T30 it was predominantly located in endosomal-like structures (**Figures 2G,H**). Moreover, the percentage of colocalization between M6a and clathrin was increased in HEK293 cells at T30 (16%) compared with cells at T0 (8%) (**Figure 2H** and Supplementary Table S1). These results suggest that clathrin mediates M6a endocytosis through the 251YEDI254 motif within its C-tail.

### Endocytosed M6a Is Sorted to Both Recycling and Degradative Compartments

Transferrin (Tf) internalization depends on the binding of TfR to AP-2 followed by formation of clathrin-coated vesicles (McMahon and Boucrot, 2011). To confirm whether endocytosed M6a mimics the endocytosed Tf-TfR cellular fate, we used Tf-647 conjugate to stain recycling endosomes and LysoTracker to stain acidic compartments in M6a-expressing neurons and HEK293 cells subjected to the AIA. Endocytosed M6a and endocytosed Tf share a small portion of endocytic compartments at T1 but not at T0 in neurons and HEK293 cells (**Figures 3A,B**). Also, a 20% of colocalization between the endocytosed M6a and Tf-647 in HEK293 cells at T1 was quantified (**Figure 3B** and statistical data see Supplementary Table S1). Using LysoTracker staining, endocytosed M6a colabelled with acid endosomes at T1 in both cells types (**Figures 3C,D**). To confirm this, we labeled M6a-overexpressing HEK293 cells with antibodies against human LAMP-1, which labeled late endosomes and lysosomes (LE/Lys). A colocalization between endocytosed M6a and LAMP 1-positive endosomes was found at T1 cells (**Figure 3E**). Similar results were obtained co-expressing LAMP-1GFP and M6aRFP in HEK293 cells (**Figure 3F**). Indeed, the percentage of colocalization between endocytosed M6a with LysoTracker, endogenous LAMP-1 and LAMP-1GFP was increased in HEK293 cells at T1 compared with cells at T0 (**Figures 3D,F** and Supplementary Table S1). These results suggest that M6a is targeted by CME to Tf-positive endosomes (likely representing the recycling endosome) and also to LAMP1-positive LE/Lys endosomes- (likely the degradation endosome).

### Endocytosed M6a Is Sorted to EEA1- and Rab5-Positive Early Endosomes

**FIGURE 4:**
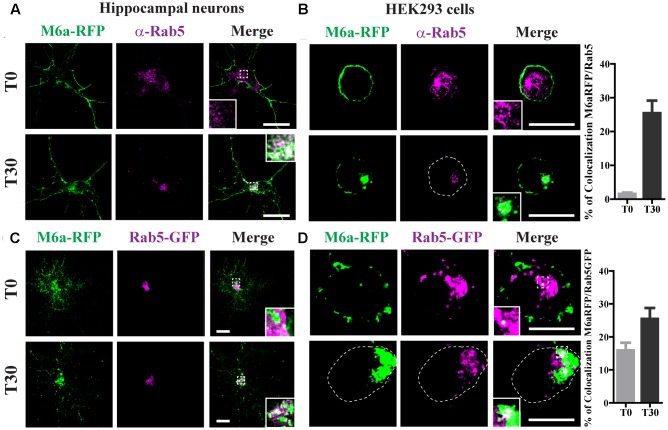
Endocytosed M6a is sorted to Rab5-positive early endosomes. M6aRFP- expressing neurons at 15 DIV and HEK293 cells were subjected to the antibody internalization assay (AIA). Representative confocal images from M6aRFP-overexpressing cells (shown in green) subjected to the AIA and labeled with anti-Rab5 (shown in magenta). Both neurons **(A)** and HEK293 cells **(B)** showed punctate colocalization between M6a and the early endosomal marker, Rab 5, at T30, as shown in the inset in white. Coexpressed M6aRFP/Rab5-GFP neurons **(C)** and HEK293 cells **(D)** were subjected to the AIA. Punctate colocalization between both expressed proteins can be seen at T0 and T30 of both types of cells (white in the inset). T0 and T30 represent the steady state and 30 min after immunouptake, respectively. The percentage of colocalization between endocytosed M6a with Rab5 and Rab5-GFP in HEK 293 cells was plotted in **(B,D)** respectively (for statistical data see Supplementary Table S1). Scale bar: 15 μm. Inset for neurons: 5 × 5 μm; inset for HEK293: 4 × 4 μm.

Following the analysis of the fate of endocytosed M6a, we evaluated the sorting into different RabGTPases and EEA1-positive endosomes. As described above, neurons at 15 DIV and HEK293 cells were transiently transfected with M6a-RFP and subjected to the AIA. Afterward, endogenous Rab5 was detected as punctate cytoplasmic staining using anti-Rab5 primary antibodies and Alexa 488-conjugate secondary antibodies (**Figure 4**). At T30, 25% of endocytosed M6a colocalized with Rab5-positive endosomes in neurons and HEK293 cells (**Figures 4A,B** and Supplementary Table S1). Similar results were found in M6a-RFP/Rab5-GFP-co-expressing hippocampal neurons and HEK293 cells. Also, the percentage of colocalization between endocytosed M6a-RFP and Rab5-GFP positive endosomes increased from 15% at T0 to 25% at T30 in HEK293 cells (**Figure 4D** and Supplementary Table S1). Sato et al. demonstrated that M6a is targeted to EEA1-positive endosomes but not to LAMP-1-positive endosomes after 2 h of incubation with other monoclonal anti-M6a antibodies (mAb 1B4) in stable M6a-HEK293 cells (Sato et al., 2011b). Thus, we labeled endogenous EEA1-positive endosomes in M6a-overexpressing cells subjected to the AIA. In concordance with that found by Sato et al., few EEA1-positive endosomes colocalized with endocytosed M6a at T30 (Supplementary Figure S3).

### Endocytosed M6a Is Sorted to Both Late and Recycling Endosomes

**FIGURE 5:**
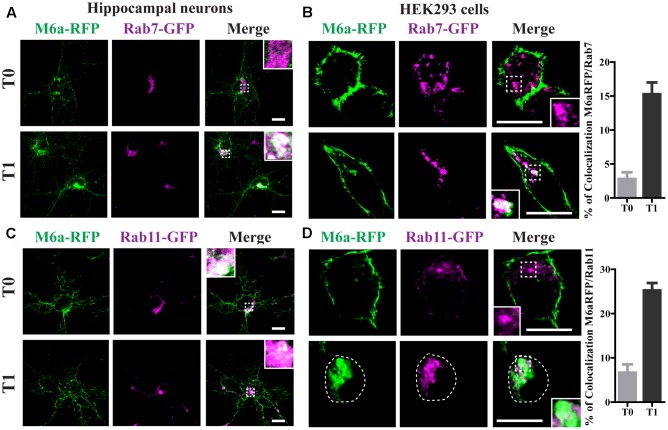
Endocytosed M6a is sorted to both late and recycling endosomes. M6aRFP/Rab7- or Rab11-coexpressing neurons and HEK293 cells were subjected to the antibody internalization assay (AIA). **(A**,**B)** Representative confocal images from M6aRFP/Rab7GFP-overexpressing cells at T0 (steady state) and T1 (1 h after immunouptake). **(C**,**D)** Representative confocal images from M6aRFP/Rab11GFP-overexpressing cells at T0 and T1. M6aRFP is shown in green and RabGTPases are shown in magenta. Conditions in which dots exhibited colocalization between endocytosed M6a and the corresponding endocytic compartments are shown in white in the inset of merge images and are plotted for HEK293 cells (for statistical data see Supplementary Table S1). Scale bar: 15 μm. Inset for neurons: 5 × 5 μm; inset for HEK293: 4 × 4 μm.

Rab7 is required for both recycling from early endosomes and for progression to Lys/LE endosomes (Vanlandingham and Ceresa, 2009). Rab11-positive endosomes regulate the trafficking of recycle vesicles containing membrane proteins to the cell surface (Takahashi et al., 2012). To confirm if endocytosed M6a is sorted to Rab7-positive late endosomes and Rab11-positive recycling endosomes we co-expressed Rab7-GFP or Rab11-GFP with M6a-RFP in hippocampal neurons at 15 DIV and HEK293 cells. A colocalization between endocytosed M6a and Rab7-GFP-positive endosomes was found at T1 in both types of cells (**Figures 5A,B**). In contrast, at T0, both fusion proteins exhibited a distinctive subcellular localization in neurons and HEK293 cells. About 15% of endocytosed M6a colocalized with Rab7-GFP in HEK293 cells at T1 (**Figure 5B** and Supplementary Table S1). In the case of Rab11, the colocalization of M6a and Rab11-positive endosomes was evident at T1 in neurons and HEK293 cells as well as at T0 in hippocampal neurons (**Figures 5C,D**). The percentage of colocalization between endocytosed M6a and Rab11-GFP positive particles increased from 6% at T0 to 25% at T1 (**Figure 5D** and Supplementary Table S1). These results allow us to speculate that 80% of the internalized M6a is targeted to CME and recycles to the cell surface via EEA1-, Rab5-, and Rab11-positive endosomes. Moreover, 15% of endocytosed M6a is targeted to Rab7-positive late compartments.

### Caveolae/Raft-Mediated Endocytosis Is Not Involved in M6a Sorting

**FIGURE 6:**
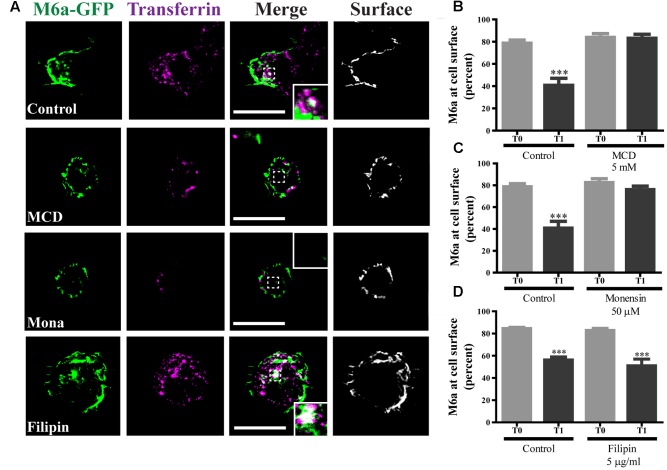
Caveolae/Raft-mediated endocytosis is not involved in M6a sorting. M6aGFP-expressing HEK293 cells were subjected to the antibody internalization assay (AIA) in the presence or absence of specific endocytic inhibitors. Methyl-β-cyclodextrin (MCD, 5 mM) and monensin (Mona, 50 μM) were used as inhibitors of clathrin-mediated endocytosis (CME). Filipin (5 μg/ml) was used as inhibitor of caveolae/raft-mediated endocytosis. Transferrin (Tf) 647-conjugate was used to track CME. **(A)** The panel shows representative confocal images of M6aRFP-HEK293 cells at T1 (1 h after immunouptake) in green, Tf in magenta and surface M6a in gray. **(B–D)** At least three independent experiments were analyzed for each condition and the percentage of M6a at the cell surface plotted as described in the Section “*Material and Methods.”* Significant differences were determined using one way ANOVA [*F*(3,36) = 23.60, *p* = 0.25] followed by Bonferroni test. **(B)** T0 vs. T1 ^∗∗∗^*p* < 0.001 (*n* = 11); **(C)** T0 vs. T1 ^∗∗∗^*p* < 0.001 (*n* = 11); **(D)** T0 vs. T1 ^∗∗∗^*p* < 0.001 (*n* = 11) and T0 vs. T1 (filipin) ^∗∗∗^*p* < 0.001 (*n* = 9). T0 represents the steady state and T1 1 h after of the immunouptake. Scale bars: 15 μm. Inset: 4 × 4 μm.

We have previously reported that part of the M6a at the cell surface of neurons and COS-7 cells is functionally associated with specific membrane (lipid) rafts (Scorticati et al., 2011). We therefore investigated whether M6a internalization only involves clathrin-dependent pathways. For that purpose, methyl-β-cyclodextrin (MCD) and monensin (Mona), which inhibit CME, and filipin, which inhibits clathrin-independent endocytosis, were used (Ceresa et al., 1998; Rodal et al., 1999; Wu et al., 2007; Winterstein et al., 2008). Representative images of control conditions are shown in Supplementary Figure S4 (T0) and those of M6a-RFP-expressing HEK293 cells at T1 treated or not with the inhibitors are shown in **Figure 6A**. As described above, under control conditions, some endocytosed M6a was colocalized with endocytosed Tf at T1 during the AIA. Tf and M6a uptake was disrupted in the presence of MCD (5 mM) and Mona (50 μM) showing total surface localization. In contrast, specific inhibition of the cholesterol-dependent pathway with filipin 5 μg/ml did not alter M6a endocytosis (dots of colocalization between M6a and Tf can be observed in the cytoplasm of HEK293 cells at T1). In addition, we quantified the surface levels of M6a for each condition as described for **Figure 2C** and found that neither MCD nor Mona modified the presence of M6a at the cell surface at T1, showing equal amounts of M6a at T0 in any of the conditions (**Figures 6B,C**). Conversely, filipin treatment did not affect M6a internalization by the monoclonal antibodies, at the period of time evaluated, showing equal amounts of M6a at the cell surface of the control cells at T1 (**Figure 6D**). These data confirmed that M6a is internalized via CME.

### Part of Endocytosed M6a Is Subjected to Degradation

**FIGURE 7:**
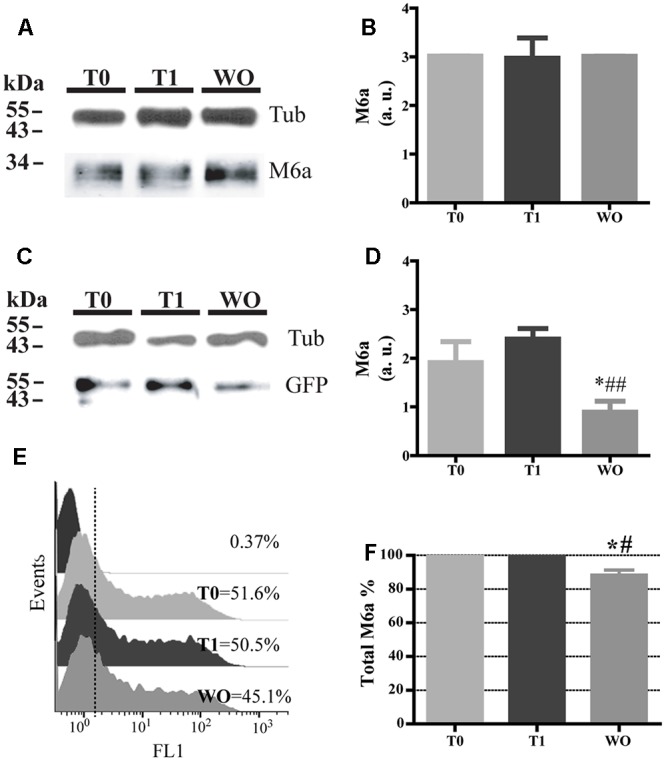
Part of the endocytosed M6a is subjected to degradation. Hippocampal neurons and M6aGFP-overexpressing HEK293 cells were subjected to the antibody internalization assay (AIA). In HEK 293 cells, the washout treatment (WO, 4 h) was done in the presence of cycloheximide to prevent new protein synthesis. Cells were collected mechanically. One aliquot was used for Western blot and in the case of transfected HEK 293 cells another aliquot was used for flow cytometry. **(A)** Representative Western blot analysis of neurons subjected to the AIA using polyclonal anti-M6a (1:1000), which recognizes its C-tail and α-tubulin (1:1000) as a loading control. **(B)** The ratio of pixel intensity of the bands corresponding to M6a/tub was quantified in three independent experiments with ImageJ and plotted as arbitrary units. **(C)** Representative Western blot analysis of lysates containing an equal percentage of transfected HEK293 cells of each condition subjected to SDS-PAGE using polyclonal anti-GFP (1:1000) and α-tubulin (1:1000) as a loading control. **(D)** Three independent experiments were quantified as in B and plotted. Significant differences were determined using one way ANOVA [*F*(2,6) = 13.33; *P* = 0.0062]. Bonferroni *post hoc* analysis reveals a significant difference between T0 vs. WO and T1 vs. W0 ^∗^*p* < 0.05, *n* = 3. **(E)** Representative flow cytometry histograms in which FL1 intensity represents the total amount of M6aGFP in each condition per cell (event). The FL1 of intact cells were used to define a positive population for each condition (less than 1%) and their percentage. **(F)** Three independent experiments in which total M6a was calculated as the ratio between FL1-positive events for each condition relativized to T0. Significant differences were determined using one way ANOVA [*F*(2,6) = 14.01, *p* = 0.0055]. Bonferroni *post hoc* analysis reveals a significant difference between T0 vs. WO ^∗^*p* < 0.05 and T1 vs. WO ^##^*p* < 0.01, *n* = 3.

Twenty percent of the endocytosed M6a was localized in LE/Lys endosomes (**Figures 3C–F**), so we next aimed to find out whether M6a can be subjected to degradation. Thus, we first performed the AIA in hippocampal neurons at 15 DIV and M6aGFP-expressing HEK293 cells. In neurons, we immunodetected the total amount of endogenous M6a at T0, T1, and WO (20 h after medium replacement) by Western blot using a polyclonal antibody against the C-tail of M6a. M6a can be displayed as a single or a double band of approximately 32–35 kDa as previously described (Fuchsova et al., 2009; Scorticati et al., 2011; Formoso et al., 2015b). In HEK293 cells, the replacement medium (WO) was supplemented with cycloheximide and maintained for 4 h to prevent the amount of M6a per cell from being modified by the new synthesis. In hippocampal neurons, no notable reduction of M6a was observed at this period of time (**Figure 7A**). We also quantified the ratio of the band intensity between the total amount of endogenous M6a and total alpha-tubulin, as loading control, by using ImageJ software, and found no significant differences in any of the conditions tested (**Figure 7B**). In M6a-HEK293 cells, the amount of M6aGFP significantly decreased at WO compared to T0 and T1 cells (**Figures 7C,D**). In the latter, an aliquot for each condition was taken to determine the total amount of M6aGFP of transfected cells by flow cytometry. **Figure 7E** shows a representative histogram plot of the fluorescence intensity (FL1) versus the number of cells (events) detected in one independent experiment. The FL1 of the non-transfected HEK293 cells were used to establish a clear division between negative and positive-GFP cells (<1% of the total cells assessed). As observed in the Western blot, T0 and T1 cells displayed equal amounts of M6aGFP per cell. Regarding the WO cells, 4 h after removal of the antibody, there was a moderate reduction of the quantity of M6aGFP-tagged protein compared to T0 and T1 cells. The values obtained from the flow cytometry in three independent experiments were plotted in **Figure 7F**. No significant differences in the protein levels were observed between M6a-expressing HEK293 cells at T0 and T1. In contrast, M6a-expressing HEK293 cells subjected to the AIA followed by medium replacement for 4 h exhibited a significant decrease in the M6a levels compared with T0 and T1 cells. Based on these last results, we speculated that the LAMP-1-positive endosomes in which endocytosed M6a was present serve as degradative organelles rather than storage compartments, as in the case of PLP (Winterstein et al., 2008).

## Discussion

The mechanisms by which M6a internalizes and recycles in the neuronal surface are particularly relevant to clarify because this glycoprotein regulates synapse formation and maintenance. In this work, we demonstrated that M6a internalization involves CME, probably through the association of AP-2 with the M6a-251YEDI254 “tyrosine based” motif located within its C-tail. Upon endocytosis, the M6a internalized through CME can be sorted back to the cell surface via a common endosomal network. In the initial sorting decision, the EEA1- and Rab5-positive early endosomes determine M6a cellular fate. Eventually, the endocytosed M6a can be sorted back to the cell surface via Rab11-positive endosomes or be sorted to the trans-Golgi network via retrograde traffic (Rab7-positive endosomes) or/and be sorted to the lysosomal compartments for degradation (LAMP-1-positive endosomes).

No ligands or natural partners which control M6a function have yet been found. Thus, there is a restriction for the discovery of M6a trafficking and signaling inside cells. Monoclonal antibodies against extracellular domains of membrane proteins are widely used to study their sorting and recycling pathways (Pula et al., 2005). For instance, a monoclonal antibody against an extracellular epitope of the p75 neurotrophin receptor has been used to track its cellular fate after agonist stimuli in PC12 cells and sympathetic neurons (Escudero et al., 2014). In the case of PLP, Winterstein et al. (2008) demonstrated that the internalization of the protein after treatment with a monoclonal antibody against its EC2 in oligodendrocytes exhibits natural endocytic behavior. In this work, we used the monoclonal antibodies against the major external loop -EC2- of M6a (M6a mAb) developed by Lagenaur et al. (1992). There is currently insufficient evidence to establish why the treatment with M6a mAb inhibits neurite outgrowth and extension in neurons and cell lines (Lagenaur et al., 1992; Formoso et al., 2015a). Here, we showed that the surface levels of M6a drastically decreased under M6a mAb treatment in hippocampal neurons. This was accompanied by a decrease in the number of synaptophysin puncta, NMDA-R1 puncta and number of synapses, without changes in the protein levels of the synaptic markers. Moreover, neurons at 15 DIV exhibited no morphological changes under antibody treatment. After 4 h of anti-M6a mAb removal, total endogenous M6a was placed at the cell surface; however, it took 20 h for total recovery of the number of synapses. The results obtained in the WO cells (**Figures 1C**,**7D,F**), which suggests that M6a is degraded, allow us to speculate that new synthesis is required to recover the normal number of synapses. These results indicate that the levels of M6a at the cell surface modified synapse formation and maintenance in neurons. Consistently, we have recently reported that overexpression of M6a wt in hippocampal neurons at 15 DIV exhibits a marked increase in the number of mature dendritic spines and synapses, whereas the overexpressed *GPM6A* SNPs variants modified neither spine density nor synapses, as if the proteins had not been expressed (Formoso et al., 2016).

Most of the sorting signals are located within cytoplasmic tails of the receptors and membrane proteins that target into the endocytic and secretory pathways. CME is an internalization mechanism in which cargo proteins contain short sequence motifs such as [D/E]XXXL[L/I] and YXXΦ, which bind AP-2, and NPXY motifs, which bind other clathrin adaptor proteins (Pandey, 2009; Cosker and Segal, 2014; Robinson, 2015). In this study, we characterized the 251YEDI254 motif within the C-tail of M6a predicted by the ELM data base, which can be recognized by mu subunit of the AP-2 complex. Our results indicate that the M6a overexpressed in HEK293 cells efficiently internalizes, recycles back to the cell surface, and is removed like endogenous M6a in primary cultures. In contrast, the substitution of tyrosine by alanine blocked the rapid protein internalization under the monoclonal stimuli in Y251A-HEK293 cells. Besides, this motif is not limited to endocytosis/recycling, since it has been associated with the targeting of transmembrane proteins to lysosomes or multivesicular bodies (Williams and Fukuda, 1990; Harter and Mellman, 1992; Marks et al., 1995). Moreover, YXXΦ motifs sorting to lysosomes have a tendency to possess acid residues at XX positions (Bonifacino and Dell’Angelica, 1999). Certainly, glutamic (E) and aspartic (D) acids are acidic amino acids that are placed within YXXΦ (YEDI) of the M6a motif. In concordance with our findings, 20% of the endocytosed M6a is located in LAMP-1-positive endosomes.

Endosomes also serve as a membrane platform for some receptors which quickly endocytose by the receptor-ligand association and continue their signaling cascade (Sorkin and von Zastrow, 2009). Conversely, it has been demonstrated that phosphorylation of the C-tails of certain membrane proteins placed within a sorting linear motif disrupts protein internalization (Bonifacino and Dell’Angelica, 1999). For instance, phosphorylation at Y165 in the YXXΦ motif of the glycoprotein cytotoxic T lymphocyte antigen-4 (CTLA-4), which recruits and activates the phosphatidylinositol-3 kinase (PI3K) pathway, inhibits the interaction with AP-2 (Shiratori et al., 1997). We have previously reported that a phosphorylation form of M6a at Y251 (Y251D), which recruits and activates the PI3K/AKT pathway, enhances neurite outgrowth in neurons and N2a cells. In addition, M6a phosphorylated mutant, Y251D, completely rescues the inhibition of neurite elongation caused by the treatment with anti-M6a mAb (Formoso et al., 2015a). The results presented here led us to speculate that both Y251 mutants (Y251A and Y251D) abolish the sorting motif, preventing M6a internalization. Moreover, in the case of the Y251D mutation, in addition to eliminating the tyrosine-based sorting motif, the phosphomimetic replacement results in constitutive activity of the signal cascade leading to neurite outgrowth.

Wu et al. (2007) and Liang et al. (2008) identified M6a as a promoter of the μ-opioid receptor targeting into the recycle network after agonist stimuli in stable MOPr-M6a-co-expressing HEK293 cells and primary cultured neurons. In both studies, the authors tracked the cell fate of M6a and MOPr by surface-labeling, *in vivo*, with anti-M6a mAb and anti- MOPr antibodies respectively before μ-opioid agonist stimulation. Thus, it seems that the authors stimulated the internalization of both MOPr and M6a at the same time. In addition, they found that M6a internalization was disrupted in the presence of the hypertonic sucrose used to inhibit CME. In agreement, in this work, M6a immunointernalization was totally blocked in the presence of monensin and methyl-β-cyclodextrin. Filipin, which affects the internalization of lipid-rafts partitioned proteins, did not modify M6a internalization. By contrast, in the presence of filipin, the AIA blocks the endocytosis of PLP. PLP is taken up by a caveolae/raft-sensitive pathway and stored in LE/Lys compartments until required for myelin compaction, showing a distinct behavior within the PLP family (Simons et al., 2000; Kramer-Albers et al., 2006; Winterstein et al., 2008). There are controversies about the use of specific inhibitors of the different endocytic/recycling pathways. Most studies focus on the concentrations and exposure time and use starved-serum conditions (Dutta and Donaldson, 2012). Since there is a consensus that TfR exclusively internalizes via CME, here we used the Tf 647-conjugate as a prototype in the inhibition experiments during the AIA.

In summary, in this work, we identified that M6a is endocytosed and recycled back to the cell membrane via CME, thus expanding the range of membrane proteins that can be subjected to CME. We also identified certain amino acids within the C-tail of M6a that are required for the endocytosis/sorting decision. We concluded that M6a reduction at the cell surface decreases hippocampal synapse numbers and that this is rescued by restoring M6a levels. This mechanism might be critical during neuronal development, pruning and/or many of the neurological disorders in which the number of synapses is affected.

## Author Contributions

The data collection and the interpretation of the results were made mostly by MG in collaboration with GA and KF. In Addition, MG prepared the figures. CS designed the experiments and conducted the study as a principal investigator. The manuscript was written by CS and critical revised by AF and commented on by all other authors. This study was mostly granted by AF and less CS.

## Conflict of Interest Statement

The authors declare that the research was conducted in the absence of any commercial or financial relationships that could be construed as a potential conflict of interest.

## Footnotes

1 https://github.com/ekatrukha/ComDet/wiki

2 http://rsbweb.nih.gov/ij/

3 http://elm.eu.org
