# Supplementary material for: The Membrane Glycoprotein M6a Endocytic/Recycling Pathway Involves Clathrin-Mediated Endocytosis and Affects Neuronal Synapses
Source: Front Mol Neurosci. 2017 Sep 20;10:296. doi: 10.3389/fnmol.2017.00296 (PMC5611492; doi:10.3389/fnmol.2017.00296)
Supplement: Supplementary file 1 [file Table_1.DOCX]

**Table S1. Quantification of endocytosed M6a colocalization with different endocytic markers in HEK293 cells**

*****Percentage of endocytosed M6a (A) colocalized with B-positive particles

**Supplementary Table S1: Quantification of endocytosed M6a colocalization with different endocytic markers in HEK293 cells:** Confocal images of HEK293 cells were analysed using ComDet Image J plugin to calculate the percentage of colocalization between endocytosed M6a with the indicated markers. The numbers were expressed as percentages. Representative images are in Fig. 2H, Fig. 3 (B, D, E and F), Fig 4 (B and D), Fig.5 (B and D) and Fig. S3B. The percentage of colocalization between two completely separate endosomal populations (Rab5-positive endosomes with LAMP1-GFP positive endosomes) was less than 3 % in T0 (2.542 ± 0.3710, n=24) and T1 (1.962 ± 0.2854, n=26) showed non-significant differences (t48=1,25, p=0.2172) .Thus, less than 3-4% of colocalization can be considered no colocalization.
