# Supplementary figures and images for: The Membrane Glycoprotein M6a Endocytic/Recycling Pathway Involves Clathrin-Mediated Endocytosis and Affects Neuronal Synapses
Source: Front Mol Neurosci. 2017 Sep 20;10:296. doi: 10.3389/fnmol.2017.00296 (PMC5611492; doi:10.3389/fnmol.2017.00296)

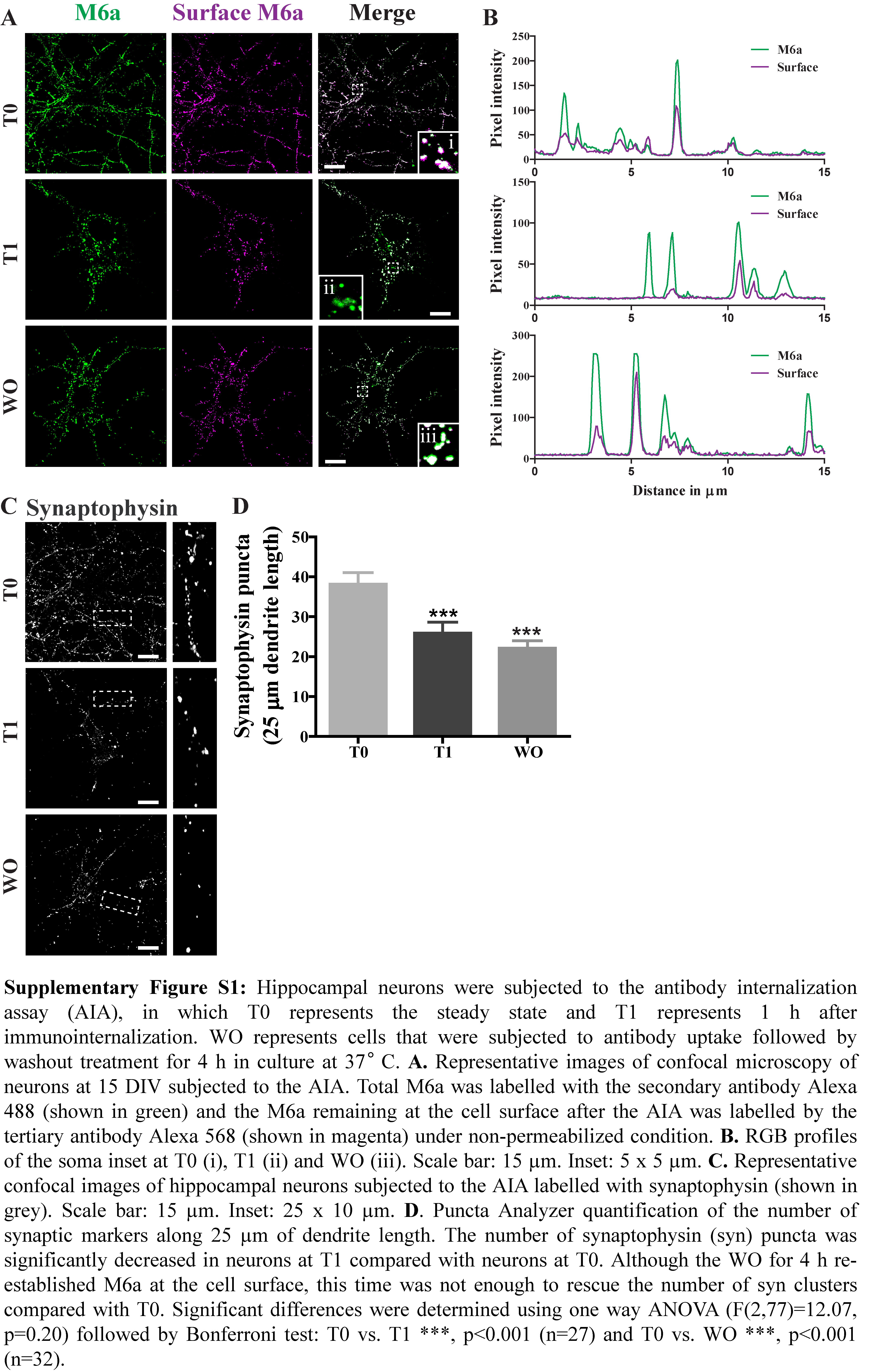

Supplement: Supplementary file 2 [file Image_1.tif]

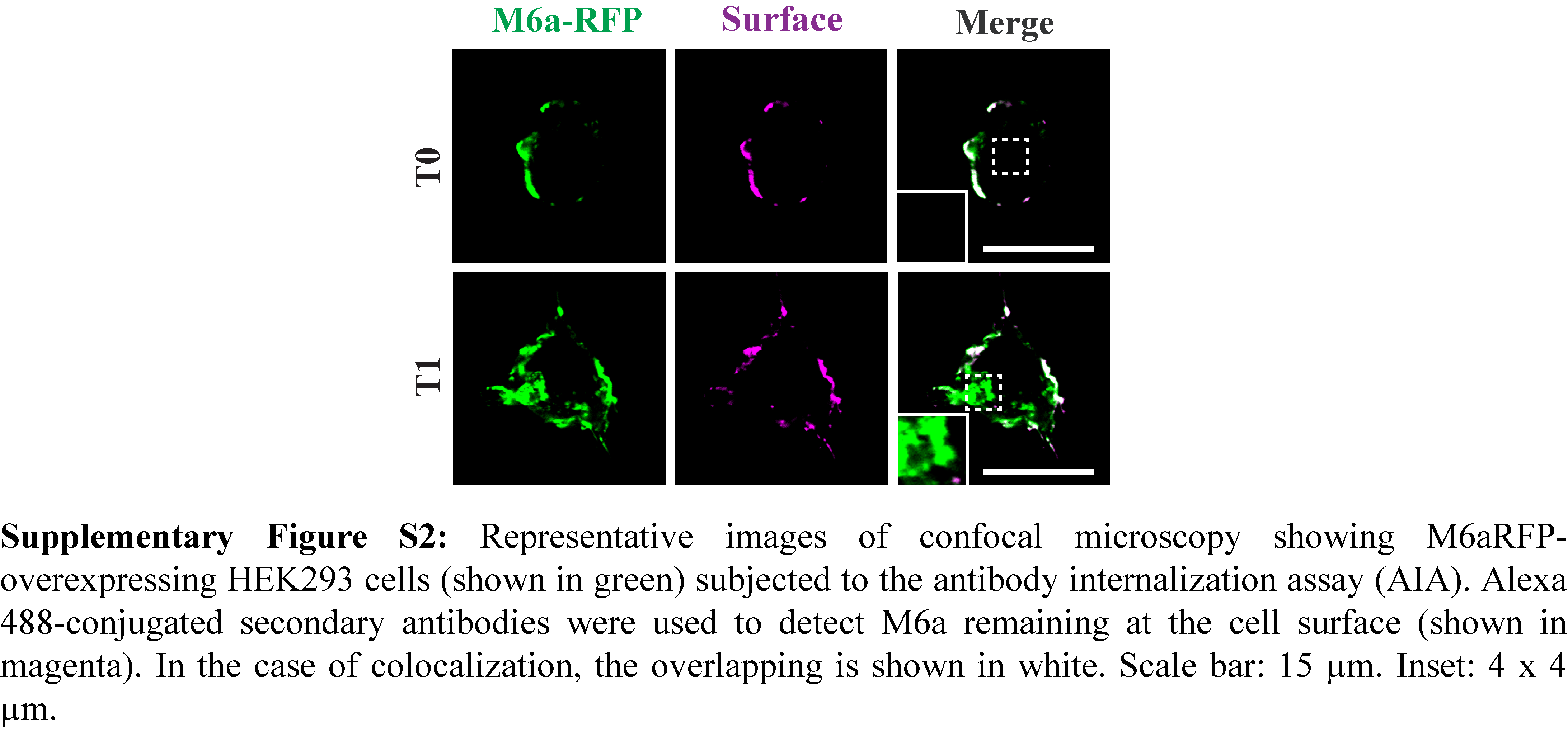

Supplement: Supplementary file 3 [file Image_2.tif]

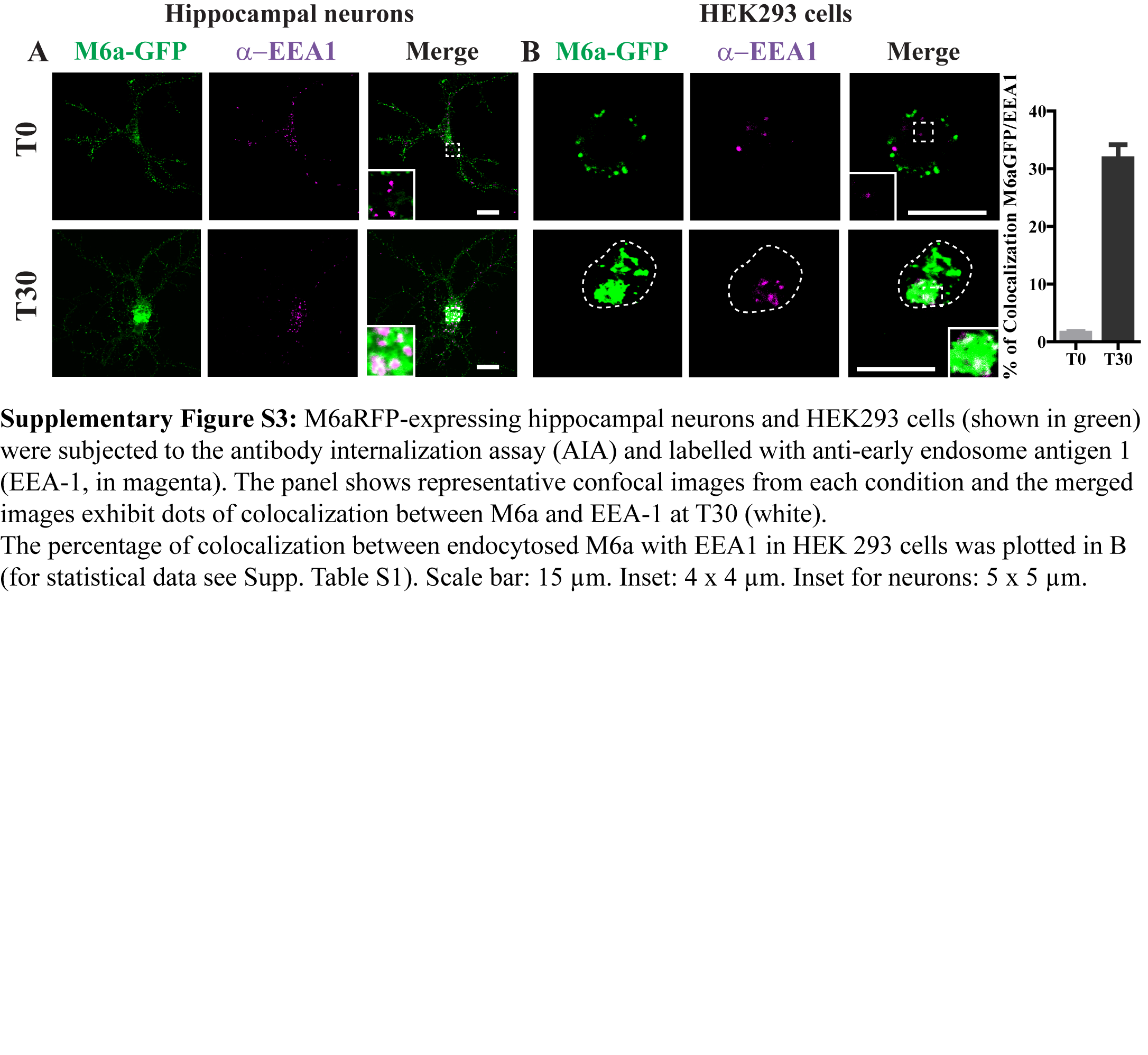

Supplement: Supplementary file 4 [file Image_3.TIF]

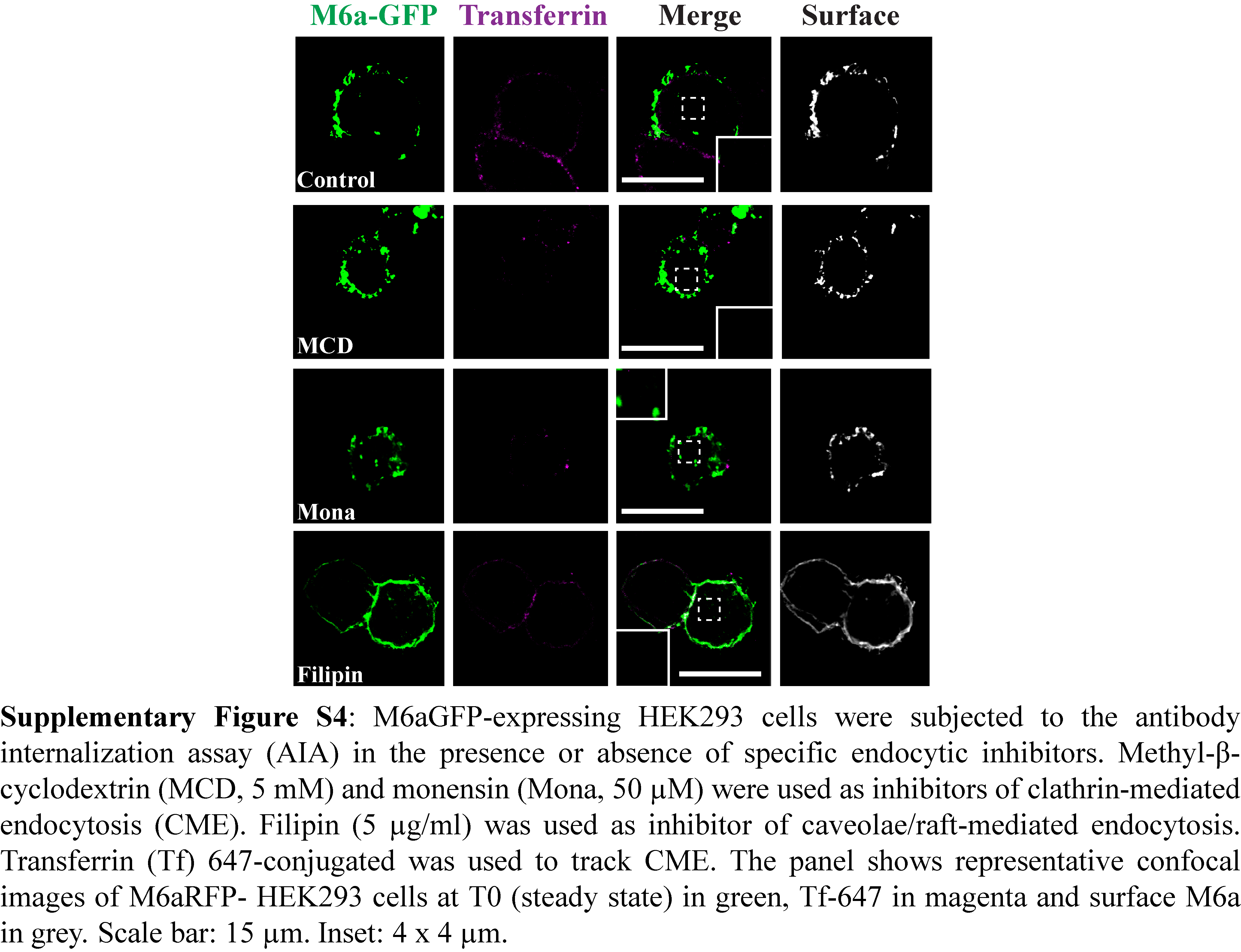

Supplement: Supplementary file 5 [file Image_4.tif]
